# Effects of Fish Oil and Dietary Antioxidant Supplementation on Bone Health of Growing Lambs

**DOI:** 10.3390/ani11010230

**Published:** 2021-01-18

**Authors:** Grzegorz Skiba, Stanisława Raj, Monika Sobol, Marian Czauderna, Paweł Kowalczyk, Eugeniusz R. Grela

**Affiliations:** 1 Department of Animal Nutrition, Polish Academy of Sciences, The Kielanowski Institute of Animal Physiology and Nutrition, Instytucka 3, 05-110 Jabłonna, Poland; s.raj@ifzz.pl (S.R.); m.sobol@ifzz.pl (M.S.); m.czauderna@ifzz.pl (M.C.); p.kowalczyk@ifzz.pl (P.K.); 2 Institute of Animal Nutrition and Bromatology, University of Life Sciences in Lublin, Akademicka 13, 20-950 Lublin, Poland; eugeniusz.grela@up.lublin.pl

**Keywords:** bone health, fish oil, carnosic acid, selenium, growing lambs

## Abstract

**Simple Summary:**

The current study investigated the bone status of growing lambs fed diets supplemented with bioactive components (fish oil, carnosic acid, SeY, and Na_2_SeO_3_) improving bone parameters. The study provides new information with regards to the positive role of bioactive components supplemented to diets for growing lambs on their femur characteristics (bone content, bone mineral density, geometry, and strength).

**Abstract:**

The aim of the present study was to assess the effects of partial replacement of rapeseed oil (RO) with fish oil (FO) combined with dietary supplementation of various antioxidants on the characteristics of lamb femur. Thirty male lambs were assigned to five dietary treatments and fed isoproteinous and isoenergetic diets for 35 days. The control diet was enriched with 3.0% RO, while the experimental diets were enriched either only with 2.0% RO and 1.0% FO or additionally with 0.1% carnosic acid, 0.1% carnosic acid and 0.35 ppm Se as selenized yeast, or 0.1% carnosic acid and 0.35 ppm Se as sodium selenite. After 35 days, the lambs were slaughtered, and the femur was dissected from the carcass of each animal and analyzed for morphometric, geometric, densitometric, and biomechanical properties. The dietary modifications, specifically the supplementation of FO and selenized yeast, significantly improved the geometric, densitometric, and biomechanical properties of lamb femur.

## 1. Introduction

In previous studies, dietary supplementation of n-3 polyunsaturated fatty acids (n-3 PUFA) improved the growth, meat fatty acid profile, fertility, and immunity of pigs [1,2], chicken [3], ruminants [4,5], and rabbits [6]. However, several of these studies used fats rich in n-3 PUFAs, primarily linseed oil ((LO) a source of α-linolenic acid (ALA, C18:3n-3)) or fish oil ((FO) a source of docosahexaenoic acid (DHA, C22:6n-3)), but tested the benefits of n-3 PUFA supplementation of animal feed irrespective of the dietary source. In some studies, n-3 PUFA supplementation of diets improved the fatty acid profile as well as degree of bone mineralization and bone strength in monogastric animals [2,7]. Thus, dietary supplementation of n-3 PUFA in monogastric animals is beneficial for both animals (by improving their welfare) and humans (by improving the composition of animal products, such as meat). Recently, much scientific effort was put into the modification of fatty acid profile of ruminant tissues [5,8,9]. However, achieving benefits in this group of animals is rather difficult due to differences in the structure of the gastrointestinal tract. Therefore, studies on the modification of dietary fatty acid composition and its effects on ruminant tissues have been conducted using various feed supplements (e.g., oils or phytochemicals). Moreover, additional studies have been designed to reduce of bacterial lipolysis and subsequently suppress biohydrogenation and isomerisation in rumen, mainly through decreasing the enzymatic isomerization yield of linoleic acid (LA, C18:2n-6) or ALA via inhibition of ruminal bacterial isomerase activity [10,11,12].

Furthermore, Miezeliene et al. [13] proposed that Se regulates the key pathways of antioxidant defense mechanism in the body. In addition, Davis et al. [14] reported that Se is an essential constituent of selenoenzymes, which play pivotal roles in various physiological processes. Se is commonly added to animal diets in an inorganic (sodium selenite) or organic (e.g., selenized yeast) form; however, Gjerlaug-Enger et al. [15], Grela and Sembratowicz [16], and Rayman [17] demonstrated that the organic form shows greater bioavailability and is more effectively taken up by tissues. Some studies [18,19] indicated that partial replacement of RO with FO (rich in long-chain n-3 PUFA (LCPUFA)) combined with supplementation of carnosic acid and Se compounds (both organic and inorganic forms) affected rumen isomerization and biohydrogenation, further decreasing the tissue concentration of undesirable saturated fatty acids (SFA) and increasing the tissue concentration of desirable unsaturated fatty acids (UFA), particularly LCPUFA.

Moreover, according to Rozbicka-Wieczorek et al. [9], such a dietary supplementation decreased oxidative processes in the animal body. Oxidative stress disrupts bone remodeling, consequently reducing bone mass and bone density and increasing bone susceptibility to fractures [20]. In addition, some clinical studies [21,22] have shown that antioxidants play important roles in reducing inflammatory processes, which negatively affect bone turnover. Consistent with this, another study [23] in rabbits showed that the combined administration of sodium selenite with vitamins E and C (with antioxidant properties) was more effective in preventing structural alterations of bones than the use of vitamins alone.

However, the effects of partial replacement of RO with oils rich in LCPUFA, such as FO, combined with the supplementation of carnosic acid and organic or inorganic Se compounds on bone strength and mineralization in animal models, especially those other than rodents, remain unknown. It was shown that organic selenium exerts a significant influence on trace elements in the blood serum and liver tissue of lambs [24]. Increasing evidence indicates [25,26] that lambs or sheep can serve as an excellent large animal model for humans in studies on orthopaedic or dental defects, owing to advantages related to bone anatomy, formation, and biomechanical characteristics; ease of handling; and absorption of minerals and vitamins. Moreover, bone healing in many nonhuman animal species is faster than that in humans, whereas this rate is comparable in sheep and humans [27,28]. Additionally, sheep bones have been previously established as useful models for human bone turnover and remodeling [25]. In this context, it seems interesting to assess the effects of dietary supplementation of various oils combined with antioxidants (carnosic acid and organic or inorganic Se compounds) on bone characteristics in a sheep model. These analyses will advance our understanding of the association between dietary supplementation of various antioxidants and bone properties as well as the related metabolic processes. This information will also allow for better design and conduct of research using a lamb model (e.g., the role of natural dietary supplements in orthopedics, recovery after orthopedic procedures, and prevention of skeletal diseases).

To this end, we hypothesized that partial replacement of RO with FO combined with supplementation of various antioxidants (carnosic acid and Se) in diets would improve the tissue profile of n-3 PUFA, mineralization, geometry, and biomechanics of lamb femur. Therefore, the primary aim of the present study was to evaluate the effects of various dietary modifications with different fat and selenium sources on femur morphometry, cortical wall thickness (CWT), cross-sectional area (CSA), cortical index (CI), and strength in a lamb model.

## 2. Materials and Methods

### 2.1. Ethics

All experimental procedures in this study were performed in accordance with the relevant national or local ethical guidelines and were approved by the III Local Ethics Committee on Animal Experimentation of Warsaw University of Life Sciences, WULS, Poland. According to the principles of the 3Rs (replacement, reduction, and refinement), the study and experiments were designed to minimize the number of animals while maintaining high statistical power.

### 2.2. Animal Experiments

Material for research was obtained from an experiment evaluating the effects of dietary modification on the profile of biohydrogenation products, specifically conjugated fatty acids, in ruminal fluid and some tissues as well as microbiota [19]. The femur was collected, as this bone is commonly used in human osteoporosis and orthopedic research. Since densitometric measurements are commonly used in human medicine, the present study focused mainly on the state of bone mineralization owing to its great impact on bone strength. Scheme of the study is presented in Table 1.

The study was performed on 30 male Corriedale lambs. The animals were randomly stratified into five groups (*n* = 6 animals each) and individually kept in pens on rubber mats. The main experiment was performed following a three-week initial period (change in body weight from ~25 to 30 kg). During this period, the lambs were fed only a basal diet (BD) containing 36.0% meadow hay and 64.0% concentrate with soybean meal (360 g), barley (165 g), wheat starch (90 g), and a mineral–vitamin mixture (25 g). When lambs reached the body weight of 30 kg, BD was supplemented with 3.0% RO (group C); RO (2.0%) + FO (1.0%) (group E_I_); RO (2.0%) + FO (1.0%) + carnosic acid (0.1%) (group E_II_); RO (2.0%) + FO (1.0%) + carnosic acid (0.1%) + organic Se (0.35 ppm selenized yeast) (group E_III_); or RO (2.0%) + FO (1.0%) + carnosic acid (0.1%) + mineral Se (0.35 ppm sodium selenite) (group E_IV_). These feeding schemes were maintained for the following 35 days (until the lambs reached ~37.0 kg body weight). Feed chemical composition, energy content, and fatty acid concentration are presented in Table 2 and Table 3.

During both initial and experimental periods, animals had semi-ad libitum access to diets (0.85 and 1.08 kg·day^−1^, respectively), offered twice a day (7:30 a.m. and 4:00 p.m.) in equal amounts, and ad libitum access to fresh water. The control and experimental diets were isoenergetic and isonitrogenous and were constituted of (including supplements) the following contents per kilogram dry matter, according to the feeding recommendations for ruminants [29]: 17.9 MJ gross energy, 202 g crude protein, 119 g crude fiber, and 51.7 g crude fat. RO and commercial odorless FO containing high amounts of LCPUFA were purchased from Agrosol (Pacanów, Poland) and carnosic acid was purchased from Hunan Geneham Biomedical Technology Ltd. (Changsha, China). Selenized yeast (*Saccharomyces cerevisiae*) was purchased from Sel-Plex (Alltech In., Nicholasville, GA, USA) and sodium selenite was provided by Sigma-Aldrich (St. Louis, MO, USA).

At the end of the main experimental period (35 days), after 12 h of starvation, each lamb was anesthetized via intramuscular xylazine injection (2–4 mg·10 kg^−1^ body weight) and then slaughtered by exsanguination. Next, from each right half-carcass, the femur was dissected, cleaned of any remaining flesh, weighed, and frozen (−30 °C) for subsequent analyses.

### 2.3. Determination of Chemical Composition

Dry matter, nitrogen, crude ash, crude fiber, and ether extract contents of the diets were determined using the standard methods 934.01, 984.13, 942.05, 978.10, and 920.39 of the Association of Official Analytical Chemists [30], respectively. Fatty acid content of the feeds and each diet component was determined by base- and acid-catalyzed methylation, as described by Czauderna et al. [31], followed by quantification using capillary gas chromatography coupled with mass spectrometry (GC-MS), as described by Rozbicka-Wieczorek et al. [9] GCMS-QP2010 Plus EI (Shimadzu, Tokyo, Japan) equipped with a BPX70 fused silica column (120 m × 0.25 mm inner diammeter × 0.25 μm film thickness; Phenomenex, Torrance, CA, USA), a quadruple mass selective detector (Model 5973 N), and an injection port was used, with helium as the carrier gas. Fatty acid methyl esters (FAMEs) were identified by the comparison of electron ionization spectra of standards (Sigma, St. Louis, MO, USA) and the NIST 2007 reference mass spectra (National Institute of Standard and Technology, Gaithersburg, MD, USA). All FAME analyses were based on total ion current chromatograms and/or selected ion monitoring chromatograms.

### 2.4. Densitometric Measurements of the Femur

Dual-energy X-ray absorptiometric scans of the femur were obtained using the XR-800TM (Norland Medical Systems, CooperSurgical, Fort Atkinson, WI, USA) densitometer scanner according to the manufacturer’s protocol of scanning and analysis (research-scan type). A quality assurance test was performed every day to verify the stability of the system calibration (control scans). Moreover, the system was calibrated daily using the Quality Control Phantom and Quality Assurance Calibration Standard (Norland Medical Systems). Specimens for scanning were thawed at 23 °C for 12 h prior to use. During scanning, the right femur was positioned horizontally, with the femoral head facing upwards and the condyles facing downwards, and then scanned from the distal to proximal end. All scans were performed in triplicate to avoid bone rotation, as inconsistencies in the orientation can hamper the accuracy of test results. To ensure consistency, all scans were performed by the same operator. Bone mineral content (BMC) and bone mineral density (BMD) were recorded.

### 2.5. Three-Point Bending Test of the Femur

Following dual-energy X-ray absorptiometry, a three-point bending test was performed using a TA-HDi texture analyzer (Stable Micro Systems Ltd., Goaldming, UK) to determine the biomechanical properties of the right femur, as described by Ferretti et al. [32]. The distance between bone supports was set at 40% of the femur length, and the measuring head loaded bone samples at the mid-shaft at a constant speed of 50 mm·min-1. Maximum strength (MS) and maximum elastic strength (MES) of the bone were determined.

### 2.6. Geometric Measurements of the Femur

Geometric properties of each femur were determined based on the measurements of horizontal and vertical diameters (both external and internal) using an electronic ruler after cutting the bone. CWT (mm), CSA (mm^2^), and CI (%) were determined using the following mathematical formulae:

$$Cortical\;wall\;thickness\;\left(CWT\right)=\frac{\left[\left(V+H\right)-\left(v+h\right)\right]\;}{4}\;Cross-section\;area\;\left(CSA\right)=\frac{\pi \times \left[\left(H\times V\right)-\left(h\times v\right)\right]}{4}\;Cortical\;index\;\left(CI\right)=\left(\left(\frac{H-h}{H}+\frac{V-v}{V}\right)/2\right)\times 100$$

where V is the vertical external diameter (mm), H is the horizontal external diameter (mm), v is the vertical internal diameter (mm), and h is the horizontal internal diameter (mm).

### 2.7. Statistical Analysis

Statistical analyses were performed using Statistica (version 12, StatSoft, Tulsa, OK, USA). The examined bone characteristics in different groups are presented as mean values, with statistical errors pooled as standard error. Results were analyzed with one-way ANOVA for orthogonal data. When the *F* ratio was significant, Tukey test was used to determine differences between groups. Statistical significance was set at *p* < 0.05. With an α level of 0.05, the power established at 80%, and an effect size of 0.75, the required total sample size was 30 (i.e., *n* = 6 per group). The hypothesized effect size of 0.75 was established based on descriptive statistics of a previous study [2].

## 3. Results

The experimental factors did not affect the growth rate of lambs. There were no significant differences in body weight across lamb groups (30.5 ± 0.5 and 37.3 ± 0.60 kg at the beginning and end of the main experiment, respectively).

Femur mass, length, and CWT did not differ across groups (mean 135 g, 17.24 cm, and 3.17 mm, respectively Table 4).

Femur CSA did not differ between groups C, E_II,_ and E_IV_ (mean 149 mm^2^); however, values in groups E_III_ and E_I_ did not differ (mean 157 mm^2^) and were greater than those in C, E_II_, and E_IV_ (*p* = 0.0461). The highest femur CI was recorded in group E_III_ (39.4%; *p* = 0.0315), compared with groups C, E_I_, E_II,_ and E_IV_ (mean 35.5%). The highest femur BMC was recorded in group E_III_ (37.20 g), followed by group E_II_ (35.4 g), while the lowest BMC was recorded in groups C, E_I,_ and E_IV_ (mean 34.4 g; *p* = 0.0003). Although values in groups E_II_ and E_III_ did not differ significantly, values in group E_II_ did not differ significantly from C, E_I,_ and E_IV_. The highest femur BMD was recorded in group E_III_ (0.768 g/cm^−2^). This value was greater compared with groups C, E_II_, E_I_, and E_IV_ (mean 0.713 g/cm^2^). Femur MS was recorded in the following order (*p* = 0.0002): group E_III_ (281 N) > E_I_ (261 N) > E_II_ (257 N) > E_IV_ (mean 247 N) > C (245 N). However, values in groups C, E_I_, E_II,_ and E_IV_ did not differ significantly. Femur MES followed the same order as MS (*p* = 0.0006).

## 4. Discussion

In the present study, the energetic and nutritive values of diets as well as feed intake was equalized in all experimental groups. Thus, the diets offered to lambs in a particular group differed only in terms of the content of LCPUFA in fat source of supplements. Previous studies on the modification of fatty acid composition of diets were designed to reduce bacterial lipolysis and subsequent biohydrogenation and isomerization in the rumen [10,12].

Previous studies using pigs’ [2] and rats’ [33] models indicated that dietary supplementation of n-3 PUFAs improved bone mineralization and strength. In the present study, we also found a positive effect of partial replacement of RO (a source of LA) with FO (a source of eicosapentaenoic acid C20:5n-3-EPA and DHA) on the tested bone parameters. In the body, elongase and desaturase convert LA to arachidonic acid (AA) [34]. However, both EPA and AA are substrates for the production of eicosanoids, particularly prostaglandins (PGEs). PGE_2_ is a product of polyunsaturated n-6 fatty acids (n-6 PUFA), PGE_3_ is a product of n-3 PUFA, and these PGEs exert antagonistic effects. PGE_2_ exerts inflammatory effects, whereas PGE_3_ exerts anti-inflammatory effects. The inflammatory effects of PGE_2_ and other inflammatory cytokines (e.g., interleukin-1 (IL-1), interleukin-6 (IL-6), and tumor necrosis factor-α (TNF-α)) suppress bone formation and enhance bone resorption [35], possibly through upregulating the RANKL (Receptor Activator for Nuclear Factor *κ* B Ligand) [36].

However, n-3 PUFA inhibits this reaction [37] and promotes osteoblastogenesis in the bone marrow [38] and bone formation [39]. Interestingly, in chicks’ [35] and piglets’ [40] models, diets rich in ALA did not affect femur morphology, biomechanical properties, and bone mineral content and density compared with diets rich in LA or SFA. This can be explained by the mammalian fatty acid metabolism: Limited desaturases are available in LCPUFA synthesis, leading to low endogenous EPA and DHA levels [34]. Elevated PGE_3_ synthesis also alters cell membrane structure and fluidity, which facilitates vitamin D permeation, thus playing a crucial role in the active transport of Ca across the cell membrane [41,42]. Therefore, if the EPA and DHA contents of the diet exceed the AA content, less substrate is available for eicosanoid synthesis from AA. Thus, this fatty acid composition of the diet positively affects bone health.

Carnosic acid is a major bioactive component of rosemary (*Rosmarinus officinalis*) leaves and has been reported to show antioxidant and anti-inflammatory properties [43,44,45]. Moreover, it has anti-cancer properties, provides protection to neurons, and regulates cytoprotective responses to oxidative and electrophilic stress as well as enhancing bone formation and inhibiting bone resorption [46]. The above-cited authors hypothesized that although carnosic acid is degraded in the body, its inhibitory effects on the formation of multinucleated osteoclasts would support the osteoblastic differentiation. According to these authors, inhibition of osteoclast formation is the key action of carnosic acid that improves bone health. Unfortunately, however, bone metabolism at the cellular level was not studied in the present study; thus, we cannot prove this hypothesis. Regarding the effects of carnosic acid on bone mineralization, our results indicated that the supplementation of lamb diets with this acid improved bone mineral content and bone mineral density to similar extents as did the partial replacement of RO with FO.

Se regulates the key pathways related to antioxidant defense mechanisms in all tissues by controlling glutathione metabolism through major Se-containing antioxidant enzymes. Moreover, Se is an essential nutrient, which plays pivotal roles in various physiological processes as an essential constituent of nearly 25 selenoenzymes, in which it is present as the selenoamino acid selenocysteine [16,47]. Se is commonly added to animal diets in an inorganic form (sodium selenite). However, owing to low bioavailability [15], much of inorganic Se is excreted from the body and is thus not effectively taken up by tissues, including bones [17]. Moreover, this form of Se may exert pro-oxidant and even toxic effects, particularly at high levels [48,49]. Therefore, recently, interest in the organic form of Se has increased because of its better absorption and greater biological effectiveness in pigs [50], broiler chickens [51], beef cattle [52], and laying hens [53]. In a study on rabbits, Ebeid et al. [54] found that bioactive compounds (e.g., organic Se) positively affects body metabolism. They hypothesized that the mechanism of action of these bioactive compounds is based on the mitigation of oxidative stress, which consequently protects UFA from peroxidation damage, and these UFAs can then be effectively taken up by soft tissues. Thus, similar effects are likely produced in bone, as evidenced by the positive effects of n-3 PUFA on bone parameters in a previous study on rats [33].

In the present study, we found a positive effect of simultaneous administration of a mixture of RO (a source of n-6 PUFA with pro-oxidative properties) and FO (a source of n-3 LCPUFA with antioxidative properties) with carnosic acid and organic Se on the tested bone parameters: Geometric, biomechanical, and densitometric parameters of femur in lambs that received this diet were improved compared with those of lambs that received other diets. The mixture of such bioactive compounds likely acted synergistically on the metabolism of fatty acids in the rumen as well as on their accumulation in the tissues of lambs, including the skeleton.

To the best of our knowledge, no study in the literature has explored the effects of simultaneous administration of carnosic acid and organic Se with a mixture of RO and FO on skeletal metabolism, bone mineralization, and bone strength in ruminants. The present study demonstrated that a diet containing a mixture of RO and FO enriched with carnosic acid and organic Se improved the tested parameters of lamb femur. Based on previous reports [55], we believe that this effect was caused by the greater efficacy of organic Se than that of inorganic Se in increasing the content of this element in tissues. Inorganic Se is retained in the body for a short period, and only small amounts of Se are incorporated into selenoproteins, while the majority is excreted through urine. Studies on rabbits [56], bulls [57], and pigs [58] have demonstrated greater accumulation of organic Se in meat tissues. Other studies have also shown that Se is an essential dietary nutrient, which plays vital roles in bone health through promoting bone formation during bone turnover [59] and reducing the risk of bone fracture in both animals [26] and humans [59,60]. This can be explained by the fact that Se regulates a major part of the antioxidant defense mechanism by controlling glutathione metabolism through the key Se-containing antioxidant enzymes glutathione peroxidase and thioredoxin reductase [13,60]. In turn, glutathione peroxidase protects the integrity of unsaturated bonds in membrane phospholipids by preventing damage caused by free radicals, which can initiate lipid peroxidation [61]. Therefore, these properties of Se (and other antioxidants) were believed to protect body cells from the imbalance between oxidants and antioxidants and subsequent oxidative stress, which is considered to be the primary pathogenesis of skeletal disorders (e.g., low bone mineral density or decrease in bone mass) that make bones more prone to fractures [21,62].

In summary, a large part of the function of maintaining the growth and health of bones is attributed to the balance between osteoclast and osteoblast activities, which regulate bone remodeling. Recent evidence has shown that bioactive substances (e.g., LCPUFA, carnosic acid, and organic Se), including those contained in the diet, play crucial roles in maintaining normal bone remodeling processes and protecting bone health. They prevent and/or relieve oxidative stress, inflammation, and changes in cell membrane structure and fluidity, thereby inhibiting osteocyte apoptosis and mitigating osteoclast activity to ultimately increase osteoblast activity and osteogenesis. Thus, such compounds may be used as dietary supplements for maintaining bone health, preventing skeletal diseases, and facilitating recovery following orthopedic procedures.

## 5. Conclusions

The present study indicated that dietary bioactive components may improve bone health by promoting bone mineralization in lambs. Partial replacement of RO with FO combined with dietary supplementation of carnosic acid and organic Se improved the geometric, densitometric, and biomechanical properties of lamb femur.

## Figures and Tables

**Table 1:** Scheme of the study (diet and supplements’ consumption by particular group of lambs during main experiment).

Group/Diet	Preliminary (3 Weeks)	Main Experiment (5 Weeks)					
		Diet	Supplement				
	Diet		RO (%)	FO (%)	Carnosic Acid (%)	SeY (ppm)	Na_2_SeO_3_ (ppm)
C	BD	BD	3.0	-	-	-	-
E_I_	BD	BD	2.0	1.0	-	-	-
E_II_	BD	BD	2.0	1.0	0.1	-	
E_III_	BD	BD	2.0	1.0	0.1	0.35	-
E_IV_	BD	BD	2.0	1.0	0.1	-	0.35

C: animals fed basal diet + 3.0% RO; E_I_: animals fed basal diet + 2.0% RO + 1.0% FO; E_II_: animals fed basal diet + 2.0% RO + 1.0% FO + 0.1% carnosic acid; E_III_: animals fed basal diet + 2.0% RO + 1.0% FO + 0.1% carnosic acid + 0.35 ppm SeY; E_IV_: animals fed basal diet + 2.0% RO + 1.0% FO + 0.1% carnosic acid + 0.35 ppm Na_2_SeO_3_; SeY: selenized yeast *(Saccharomyces cerevisiae*); RO: rapeseed oil; FO: fish oil; BD: basal diet (1 kg included meadow hay 360 g, concentrate consisting of soybean meal 360 g, barley 165 g, wheat starch 90 g, and mineral–vitamin mixture 25 g); mineral–vitamin mixture supplied per kg of diet: g: Ca 285, P 16, Na 56, Fe as sulphate 1, Cu as sulphate 0.5, Mn as sulphate 5.8, Zn as sulphate 7.5; mg: Co as carbonate 42, I as iodate 10, Se as selenite 6; and IU (international units): vitamin A 500,000, vitamin D_3_ 125,000, vitamin E as α-tocopherol 25,000.

**Table 2:** Chemical composition and energy values in the ingredients of diets.

Item	Meadow Hay	Concentrate		
		Barley Ground	Soybean Meal	Wheat Starch
Dry matter (DM), %	88.4	87.6	89.7	87.3
In DM, %				
crude protein	9.50	9.94	41.8	0.90
crude fibre	27.3	2.87	4.34	-
crude fat	3.40	2.50	2.25	0.09
crude ash	4.85	1.84	6.16	0.12
Gross energy, MJ/kg DM	17.1	16.3	17.8	16.7

**Table 3:** Long-chain fatty acid concentration (g/kg) in the ingredients and diets fed to animals.

Fatty Acids	Ingredients				Period of the Study		
					Preliminary	Main Experiment (Diet/Group)	
	Concentrate	Meadow hay	RO	FO	BD	C	E_I_, E_II_, E_III_, E_IV_
C18:2 n-6 (LA)	29.2	13.1	282.0	115.0	20.0	28.5	26.84
C18:3 n-3 (ALA)	1.01	4.18	38.5	21.0	2.04	3.19	3.02
C20:5 n-3 (EPA)	nd	nd	nd	6.79	nd	nd	0.07
C22:5 n-3 (DPA)	nd	nd	nd	1.56	nd	nd	0.02
C22:6 n-3 (DHA)	nd	nd	nd	26.6	nd	nd	0.27

C: animals fed basal diet + 3.0% RO; E_I_: animals fed basal diet + 2.0% RO + 1.0% FO; E_II_: animals fed basal diet + 2.0% RO + 1.0% FO + 0.1% carnosic acid; E_III_: animals fed basal diet + 2.0% RO + 1.0% FO + 0.1% carnosic acid + 0.35ppm SeY; E_IV_: animals fed basal diet + 2.0% RO + 1.0% FO + 0.1% carnosic acid + 0.35 ppm Na_2_SeO_3_; SeY: selenized yeast (*Saccharomyces cerevisiae*); RO: rapeseed oil; FO: fish oil; BD: basal diet (1 kg included meadow hay 360 g, concentrate consisting of soybean meal 360 g, barley 165 g, wheat starch 90 g, and mineral–vitamin mixture 25 g); mineral–vitamin mixture in the diet provided supplied per kg of diet: g: Ca 285, P 16, Na 56, Fe as sulphate 1, Cu as sulphate 0.5, Mn as sulphate 5.8, Zn as sulphate 7.5; mg: Co as carbonate 42, I as iodate 10, Se as selenite 6; and IU (international units): vitamin A 500,000, viamin D3 125,000, vitamin E as α-tocopherol 25,000; nd: not determined.

**Table 4:** Femur morphometric, geometric, densitometric, and biomechanical properties at the end of the study in lambs.

Item	Group/Diet					SEM	*p*-Value
	C	E_I_	E_II_	E_III_	E_IV_		
Mass, g	136	137	135	135	132	4.09	0.9491
Length, cm	17.3	17.4	17.2	17.4	16.9	0.20	0.3572
CWT, mm	3.16	3.21	3.11	3.26	3.13	0.104	0.8534
CSA, mm^2^	149 ^a^	154 ^a,b^	149 ^a^	160 ^b^	148 ^a^	4.819	0.0461
CI, %	34.7 ^a^	35.9 ^a^	35.3 ^a^	39.4 ^b^	36.0 ^a^	1.281	0.0315
BMC, g	34.3 ^A^	35.1 ^A^	35.4 ^A,B^	37.2 ^B^	33.9 ^A^	0.618	0.0003
BMD, g/cm^2^	0.703 ^A^	0.713 ^A^	0.725 ^A^	0.768 ^B^	0.710 ^A^	0.009	0.0001
MES, N	182 ^A^	190 ^A^	188 ^A^	216 ^B^	183 ^A^	5.101	0.0006
MS, N	245 ^A^	261 ^A^	257 ^A^	281 ^B^	247 ^A^	5.509	0.0002

C: animals fed basal diet + 3.0% RO; E_I_: animals fed basal diet + 2.0% RO + 1.0% FO; E_II_: animals: basal diet + 2.0% RO + 1.0% FO + 0.1% carnosic acid; E_III_: animals fed basal diet + 2.0% RO + 1.0% FO + 0.1% carnosic acid + 0.35 ppm SeY; E_IV:_ animals fed basal diet + 2.0% RO + 1.0% FO + 0.1% carnosic acid + 0.35 ppm Na_2_SeO_3_; SeY: selenized yeast (*Saccharomyces cerevisiae*); RO: rapeseed oil; FO: fish oil; CWT: cortical wall thickness; CSA: cross-sectional area; CI: cortical index; BMC: bone mineral content; BMD: bone mineral density; MES: maximum elastic strength; MS: maximum strength; SEM: standard error of the means; ^A,B^ mean values within a row with unlike superscript letters were significantly different at *p* < 0.01; ^a,b^ mean values within a row with unlike superscript letters were significantly different at *p* < 0.05.
